# A Novel Pyroptosis-Associated Long Non-coding RNA Signature Predicts Prognosis and Tumor Immune Microenvironment of Patients With Breast Cancer

**DOI:** 10.3389/fcell.2021.727183

**Published:** 2021-09-20

**Authors:** Liqin Ping, Kaiming Zhang, Xueqi Ou, Xingsheng Qiu, Xiangsheng Xiao

**Affiliations:** ^1^State Key Laboratory of Oncology in South China, Department of Medical Oncology, Sun Yat-sen University Cancer Center, Collaborative Innovation Center for Cancer Medicine, Guangzhou, China; ^2^State Key Laboratory of Oncology in South China, Department of Breast Oncology, Sun Yat-sen University Cancer Center, Collaborative Innovation Center for Cancer Medicine, Guangzhou, China; ^3^Department of Radiation Oncology, Sun Yat-sen Memorial Hospital, Sun Yat-sen University, Guangzhou, China

**Keywords:** breast cancer, pyroptosis, lncRNAs, prognosis, tumor infiltrating lymphocytes, immune checkpoints

## Abstract

**Background:** Pyroptosis, a kind of programmed cell death characterized by the rupture of cell membranes and the release of inflammatory substances, plays an important role in the occurrence and development of cancer. However, few studies focus on the pyroptosis-associated long non-coding RNAs (lncRNAs) in breast cancer (BC). The prognostic value of pyroptosis-associated lncRNAs and their relationship with tumor microenvironment (TME) in BC remain unclear. The purpose of this study was to explore the prognostic role of pyroptosis-associated lncRNAs and their relationship with TME in BC.

**Methods:** The transcriptome data and clinical data of female BC patients were downloaded from The Cancer Genome Atlas (TCGA) database. A total of 937 patients were randomly assigned to either training set or validation set. A pyroptosis-associated lncRNA signature was constructed in the training set and verified in the validation set. Functional analysis and immune microenvironment analysis related to pyroptosis-associated lncRNAs were performed. A nomogram based on the risk score and clinical characteristics was established.

**Results:** A 9-pyroptosis-associated lncRNA signature was constructed to separate BC patients into two risk groups. High-risk patients had poorer prognosis than low-risk patients. The risk score was proven to be an independent prognostic factor by multivariate Cox regression analysis. Function analysis and immune microenvironment analysis showed that low-risk BC tended to be an immunologically “hot” tumor. A nomogram was constructed with risk score and clinical characteristics. Receiver operating characteristic curve (ROC) analysis demonstrated credible predictive power of the nomogram. The area under time-dependent ROC curve (AUC) reached 0.880 at 1 year, 0.804 at 3 years, and 0.769 at 5 years in the training set, and 0.799 at 1 year, 0.794 at 3 years, and 0.728 at 5 years in the validation set.

**Conclusion:** We identified a novel pyroptosis-associated lncRNA signature that was an independent prognostic indicator for BC patients. Pyroptosis-associated lncRNAs had potential relationship with the immune microenvironment and might be therapeutic targets for BC patients.

## Introduction

Breast cancer (BC) is the most common malignant tumor in the world (Sung et al., 2021), and it is a tumor with quite strong heterogeneity (Faria et al., 2021). According to PAM50, BC was classified into five subtypes (i.e., luminal A, luminal B, Her2-enriched, normal-like, and basal-like) (Pu et al., 2020). Even so, patients with the same molecular types and clinical characteristics have different prognosis and different responses to chemotherapy or immunotherapy (Tekpli et al., 2019), suggesting that there are still subtle factors influencing its prognosis and response to treatment.

Pyroptosis is an inflammatory form of programmed cell death mediated by gasdermins (GSDMs; Xia et al., 2019). Inflammasomes activate caspase-1/4/5/11 to cleave GSDMs, and the cleaved GSDMs are transported to the cell membrane to form cellular pores leading to cell swelling and death (Fang et al., 2020). Pyroptosis plays an important role in killing cancer cells. The mechanism of some chemotherapy drugs to kill cancer cells is partly dependent on pyroptosis. It has been proven that etoposide (Wang Y. et al., 2017), paclitaxel, and cisplatin (Zhang et al., 2019) can induce pyroptosis in cancer cells with high expression of gasdermin E (GSDME). In the process of pyroptosis in cancer cells, chemokines and tumor-associated antigens (TAAs) are released to stimulate an antitumor immune response and inhibit the progression of cancer (Garg and Agostinis, 2017). On the other hand, part of the mechanism by which immune cells kill cancer cells depends on inducing pyroptosis. It has been reported that natural killer (NK) cells and cytotoxic T lymphocytes could kill cancer cells by releasing granzyme A (GZMA) to cleave GSDMB (Zhou et al., 2020) and granzyme B (GZMB) to directly cleave GSDME to activate pyroptosis in cancer cells (Zhang et al., 2020). It is suggested that there is a close relationship between pyroptosis and antitumor immunity. Immune checkpoint blockade (ICB) therapy is an effective therapeutic strategy that relies on antitumor immunity in various cancers. However, its efficacy in BC is limited (Nanda et al., 2016; Adams et al., 2019). It has been proven that the tumor-infiltrating lymphocytes (TILs) in the BC are often poor, which makes most BC known as “cold” tumor (Thorsson et al., 2018; Tekpli et al., 2019). How to increase the TILs of the tumor microenvironment (TME) and transform it into “hot” tumor is of great significance for improving the efficacy of ICB therapy in BC. Because there is a close relationship between pyroptosis and antitumor immunity, exploring the role of pyroptosis in BC and pyroptosis-related molecules will help us investigate strategies to transform “cold” BC into “hot” BC.

Long non-coding RNAs (lncRNAs) are RNAs that do not participate in protein coding but are involved in important regulatory processes, such as genomic imprinting, chromatin modification, transcriptional activation, transcriptional interference, and intranuclear transport, which are involved in the development and metastasis of BC (Liang et al., 2020). At the same time, lncRNAs can also play an important role in mediating pyroptosis. For example, it has been reported that LncRNA MEG3 could increase the expression of NLRP3 and enhance pyroptosis of endothelial cells (Zhang et al., 2018). Wang et al. (2021) revealed that lncRNA XIST promoted pyroptosis through the XIST/miR-150-5p/c-Fos axis, and Wan et al. (2020) discovered that lncRNA-H19 significantly promoted NLRP3/6 inflammasome imbalance and induced pyroptosis of microgliocyte. Furthermore, Tan et al. (2021) found that LncRNA HOTTIP could inhibit pyroptosis of ovarian cancer cells by targeting the miR-148a-3p/AKT2 axis. However, there are limited studies focusing on pyroptosis-associated lncRNAs in BC. The prognostic value of pyroptosis-associated lncRNAs and their relationship with TME in BC remain unclear.

Therefore, this study aims to identify the lncRNAs related to pyroptosis in BC and clarify the role of pyroptosis-associated lncRNAs in the TME and prognosis of BC, which not only provides important insights into the molecular and signaling pathways of pyroptosis in BC but also has important significance in transforming immunologically “cold” BC into “hot” tumor.

## Materials and Methods

### Data Acquisition and Identification of Pyroptosis-Associated lncRNAs

**FIGURE 1:**
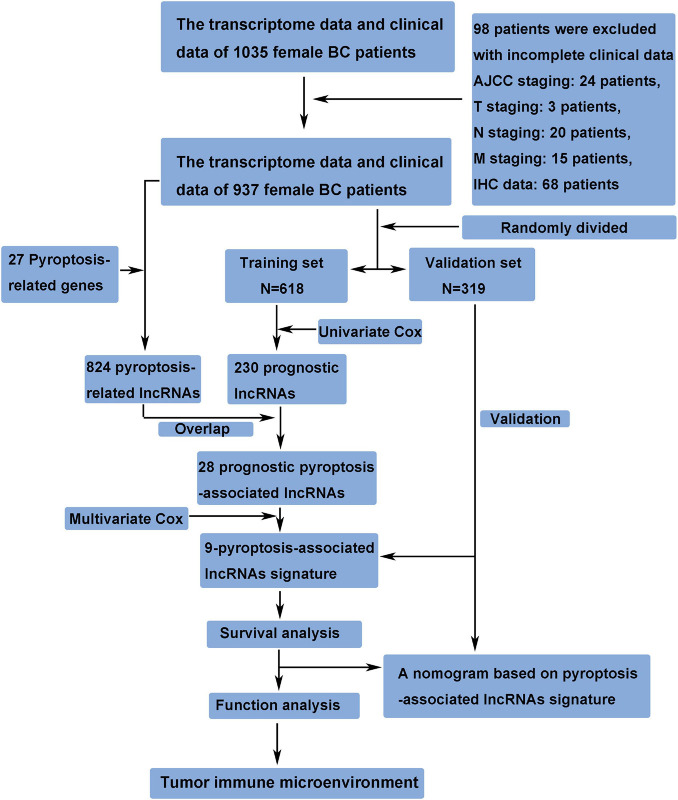
Study design and flowchart of this study.

The transcriptome data and clinical data of 1,035 female BC patients were downloaded from The Cancer Genome Atlas (TCGA) database^1^. The ensembl human genome browser GRCh38.p13 was used to distinguish the protein-coding genes and lncRNAs. Patients with incomplete clinical data were excluded from this study, and the details of these excluded patients are shown in Figure 1. The data of this study were publicly available from TCGA database, and this study was in accordance with TCGA’s publication guidelines. Pyroptosis-associated genes were downloaded from the Molecular Signatures Database version 7.4^2^. The correlation between pyroptosis-associated lncRNAs and pyroptosis-associated genes was evaluated by Pearson correlation analysis, and the pyroptosis-associated lncRNAs were identified according to the standard that the absolute value of Pearson correlation coefficient was more than 0.3 (| R| > 0.3) and the *p-*value was less than 0.05 (*p* < 0.05).

### Construction of a Prognostic Pyroptosis-Associated lncRNA Signature

A total of 937 female BC patients were randomly assigned to either training set or validation set. The prognostic lncRNAs were identified based on univariate Cox regression analysis in the training set. The overlapping lncRNAs of prognostic lncRNAs and pyroptosis-associated lncRNAs were identified as the candidate lncRNAs for the pyroptosis-associated lncRNA signature. Then, the pyroptosis-associated lncRNA signature was constructed based on multivariate Cox regression analysis and lowest Akaike information criterion (AIC) value (Vrieze, 2012). Finally, the risk score of each patient was calculated based on this prognostic signature. The formula of risk score was as follows: Risk Score = e^*sum*^ (normalized expression level of each pyroptosis-associated lncRNA × corresponding regression coefficient). Patients in the training set were separated into the high-risk group and low-risk group based on the median value of the risk score. The overall survival (OS) between the high-risk and low-risk groups was compared by Kaplan–Meier analysis.

### Validation of the Pyroptosis-Associated lncRNA Signature

The risk score of patients in the validation set was calculated according to the same formula as the training set, and patients in the validation set were separated into the high-risk group and low-risk group based on the same cutoff value as the training set. Then, Kaplan–Meier analysis was performed to compare the OS between the high-risk and low-risk groups in the validation set.

### Single Sample Gene Set Enrichment Analysis

The transcriptome data and clinical data of the Molecular Taxonomy of Breast Cancer International Consortium (METABRIC) database and GSE20685 were downloaded. The single sample gene set enrichment analysis (ssGSEA) was conducted to explore the activation level of pyroptosis pathway in BC expression profile of the METABRIC and GSE20685 database using the R package “GSVA.” Patients in the METABRIC or GSE20685 database were separated into the pyroptosis-upregulated group and pyroptosis-downregulated group based on the median value of the pyroptosis pathway score. Then, Kaplan–Meier analysis was performed to compare the prognosis between the pyroptosis-upregulated group and pyroptosis-downregulated group in the METABRIC or GSE20685 database.

### The mRNA-lncRNA Coexpression Network

The Cytoscape software version 3.7.2 was used to construct the mRNA–lncRNA coexpression network between the candidate lncRNAs and their corresponding pyroptosis-associated genes. Subsequently, the Sankey diagram was constructed to demonstrate the relationship between pyroptosis-associated lncRNAs and their corresponding genes.

### Gene Set and Function Enrichment Analysis

The differentially expressed genes between the high-risk group and low-risk group were screened with the | log2FC| ≥ 1 and the false discovery rate (FDR) <0.05 using the “edgeR” R package. The GSEA^3^ was performed to investigate the differences between the patients in high-risk group and low-risk group. The Gene Ontology (GO) analysis was performed to explore the biological processes related to the pyroptosis-associated lncRNAs, and the Kyoto Encyclopedia of Genes and Genomes (KEGG) pathway analysis was performed to identify the signaling pathways associated with the pyroptosis-associated lncRNAs.

### Analysis of Tumor-Infiltrating Immune Cells

The CIBERSORT algorithm (Newman et al., 2015) was used to calculate the proportion of each kind of tumor-infiltrating immune cells in BC samples. The results obtained by the CIBERSORT algorithm were filtered based on *p-*value <0.05. Then, the differences in each type of immune cell between the high-risk and low-risk groups were compared to assess the differences in the tumor immune microenvironment between two groups.

### Statistical Analysis

SPSS (Version 23.0) and R software (Version 3.5.3) were used to conduct statistical analyses in this study. The differences in each type of immune cell and the expression of immune checkpoint molecules between the high-risk and low-risk groups were compared by Wilcox test. Chi-square test was performed to compare the differences in clinical features. Univariate and multivariate Cox regression analyses were conducted to identify prognostic indicators of OS. The “rms” package of the R software was used to construct a nomogram including clinical features and risk score. The predictive accuracy of the nomogram was evaluated by time-dependent receiver operating characteristic curve (ROC) curve analysis. Statistical significance was defined as *p*-value <0.05, and all *p*-values were two-tailed.

## Results

### Clinical Features of Patients in Training Set and Validation Set

A total of 937 female BC patients were randomly assigned to either training set (*n* = 618) or validation set (*n* = 319) in nearly 2:1 ratio. The clinical features of all patients are shown in detail in Table 1. There were no statistically significant differences in clinical features between patients in the training set and validation set.

**TABLE 1:** Patients’ clinical features of training set and validation set.

Variables	Training set (*n* = 618)		Validation set (*n* = 319)		
	NO.	%	NO.	%	*p*-Value
Age					0.640
—≤60	347	56.1	174	54.5	
—>60	271	43.9	145	45.5	
Stage					0.352
—I	103	16.7	56	17.6	
—II	356	57.6	190	59.6	
—III	146	23.6	71	22.3	
—IV	13	2.1	2	0.6	
T stage					0.682
—T1	154	24.9	87	27.3	
—T2	368	59.5	184	57.7	
—T3	78	12.6	42	13.2	
—T4	18	2.9	6	1.9	
N stage					0.263
—N0	288	46.6	156	48.9	
—N1	209	33.8	108	33.9	
—N2	79	12.8	28	8.8	
—N3	42	6.8	27	8.5	
M stage					0.088
—M0	605	97.9	317	99.4	
—M1	13	2.1	2	0.6	
Subtype					0.488
—Non-triple negative	526	85.1	266	83.4	
—Triple negative	92	14.9	53	16.6	

### Construction of a Prognostic Pyroptosis-Associated lncRNA Signature

**FIGURE 2:**
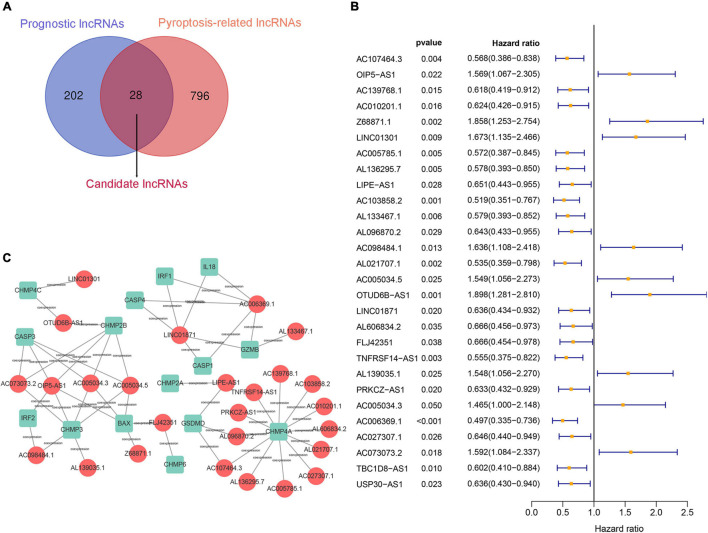
Construction of a prognostic pyroptosis-associated lncRNA signature. **(A)** Identify the overlap of lncRNAs between prognostic lncRNAs and pyroptosis-associated lncRNAs through the Venn diagram. **(B)** Results of the univariate Cox regression analysis of prognostic pyroptosis-associated lncRNAs in OS of BC patients. **(C)** LncRNA–mRNA coexpression network of pyroptosis-associated lncRNAs and corresponding genes.

First, 230 prognostic lncRNAs were identified based on univariate Cox regression analysis in the training set, and 824 lncRNAs were identified as pyroptosis-associated lncRNAs according to the coexpression relationship between lncRNAs and pyroptosis-associated genes. As shown in Figure 2A, 28 lncRNAs were overlapping lncRNAs of pyroptosis-associated lncRNAs and prognostic lncRNAs. These lncRNAs were significantly associated not only with prognosis of BC patients, but also with pyroptosis (Figures 2B,C). Finally, nine optimal lncRNAs (OIP5-AS1, Z68871.1, LINC01301, AC103858.2, AC005034.5, LINC01871, AL606834.2, TNFRSF14-AS1, and TBC1D8-AS1) were identified for the pyroptosis-associated lncRNA signature based on the lowest AIC. The risk score based on the signature was calculated according to the following formula:

risk score= e ^(0.016 × *expression level of OIP5–AS1* + 0.090^^×^^*expression level of Z68871.1* + 0.239 × *expression level of LINC01301* –^ ^0.300^ ^× *expression level of AC103858.2* + 0.054 × *expression level of AC005034. 5* –^ ^0.057 × *expression level of LINC01871* – 0.084 × *expression level of AL606834.2*^ ^– 0.123 × *expression level of TNFRSF14–AS1* – 0.323^ ^× *expression level of TBC1D8–AS1).*^

**FIGURE 3:**
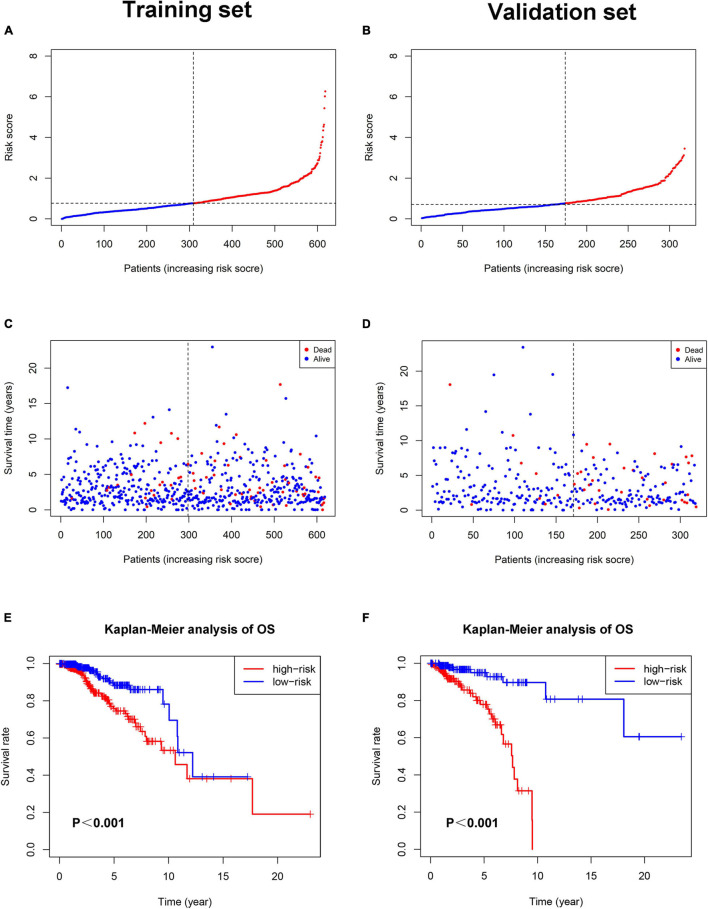
The relationship between pyroptosis-associated lncRNA signature and prognosis in the training set and validation set. The distribution of the risk scores in the training set **(A)** and validation set **(B)**. The distributions of survival status, OS, and risk score in the training set **(C)** and validation set **(D)**. **(E)** Kaplan–Meier curves show the difference in OS between the high-risk and low-risk groups in the training set. **(F)** Kaplan–Meier curves show the difference in OS between the high-risk and low-risk groups in the validation set.

**FIGURE 4:**
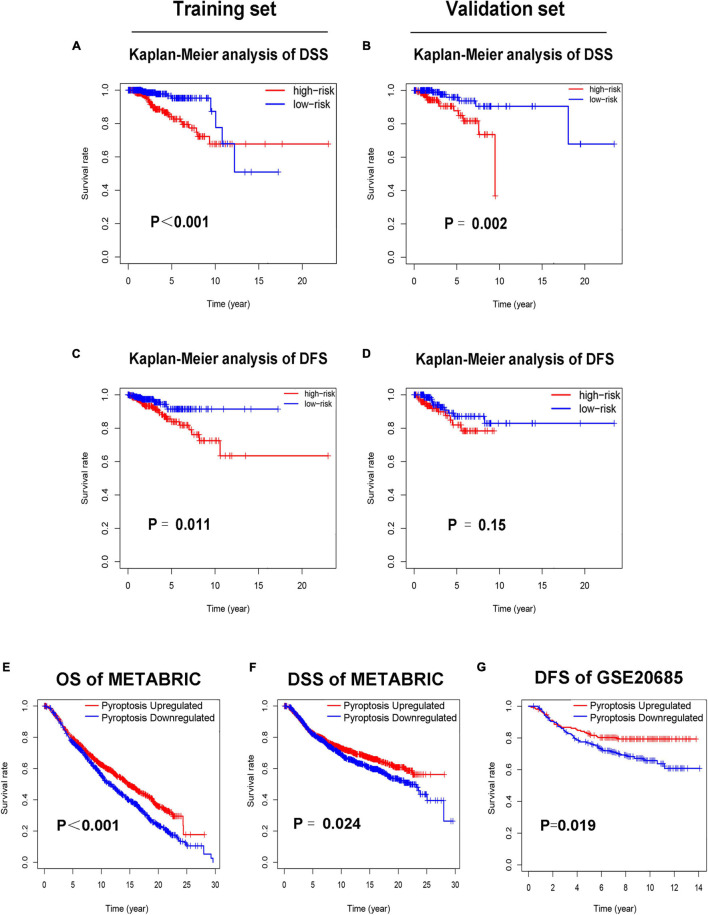
Low-risk patients had better prognosis, and pyroptosis-upregulated group had better prognosis. **(A)**. Kaplan–Meier curves of DSS between the high-risk and low-risk groups in the training set. **(B)** Kaplan–Meier curves of DSS between the high-risk and low-risk groups in the validation set. **(C)** Kaplan–Meier curves of DFS between the high-risk and low-risk groups in the training set. **(D)** Kaplan–Meier curves of DFS between the high-risk and low-risk groups in the validation set. **(E)** Kaplan–Meier curves of OS between the pyroptosis-upregulated and pyroptosis-downregulated groups in the METABRIC database. **(F)** Kaplan–Meier curves of DSS between the pyroptosis-upregulated and pyroptosis-downregulated groups in the METABRIC database. **(G)** Kaplan–Meier curves of DFS between the pyroptosis-upregulated and pyroptosis-downregulated groups in the GSE20685 database.

Each patient in the training set obtained a risk score based on the formula described above. Then, patients were separated into the high-risk group (*n* = 309) and low-risk group (*n* = 309) based on the median value of risk score (Figure 3A). The risk score was significantly related to T staging and immunohistochemical (IHC) subtype of patients with BC (Table 2). As shown in Figure 3C, BC patients with high risk score in the training set tended to die earlier. The result of the Kaplan–Meier analysis suggested that BC patients in the high-risk group had shorter OS (Figure 3E). We also analyzed disease-specific survival (DSS) and disease-free survival (DFS) of patients in these two groups, the results showed that BC patients in the high-risk group had shorter DSS and DFS than patients in low-risk group (Figures 4A,C). The 5-year DFS rate of the low-risk group was higher than that of high-risk group (91.5 vs. 83.8%). The 5-year DSS rate of the low-risk group was also higher (95.2 vs. 84.1%).

**TABLE 2:** The association between risk score and patients’ clinical features in the training set.

Variables	Low-risk group (*n* = 309)		High-risk group (*n* = 309)		
	NO.	%	NO.	%	*p*-Value
Age					0.808
—≤60	175	56.6	172	55.7	
—>60	134	43.4	137	44.3	
Stage					0.081
—I	41	13.3	62	20.1	
—II	179	57.9	177	57.3	
—III	81	26.2	65	21.0	
—IV	8	2.6	5	1.6	
T stage					0.045
—T1	68	22.0	86	27.8	
—T2	194	62.8	174	56.3	
—T3	34	11.0	44	14.2	
—T4	13	4.2	5	1.6	
N stage					0.089
—N0	131	42.4	157	50.8	
—N1	107	34.6	102	33.0	
—N2	48	15.5	31	10.0	
—N3	23	7.4	19	6.1	
M stage					0.400
—M0	301	97.4	304	98.4	
—M1	8	2.6	5	1.6	
Subtype					0.042
—Non-triple negative	272	88.0	254	72.2	
—Triple negative	37	12.0	55	17.8	

### Validation of the Pyroptosis-Associated lncRNA Signature

To verify the accuracy of the pyroptosis-associated lncRNA signature, each patient in the validation set obtained a risk score according to the same formula as the training set. Then, these patients were separated into the low-risk group (*n* = 174) and high-risk group (*n* = 145) according to the same cutoff value as the training set (Figure 3B). Consistent with the training set, BC patients with high risk score tended to die earlier in the validation set (Figure 3D). The result of the Kaplan–Meier analysis showed that BC patients in the high-risk group had poorer prognosis (Figures 3F,4B,D).

### The Activation Level of Pyroptosis Pathway Is Associated With Prognosis of BC Patients

To further confirm the relationship between pyroptosis and prognosis of BC patients, the ssGSEA was conducted to evaluate the activation level of pyroptosis pathway in BC patients of the METABRIC and GSE20685 database. Each patient in the METABRIC and GSE20685 database obtained a pyroptosis pathway score based on ssGSEA. Patients in the METABRIC or GSE20685 database were separated into the pyroptosis-upregulated group and pyroptosis-downregulated group based on the median value of pyroptosis pathway score. Then, the OS and DSS were analyzed in the METABRIC database, and the DFS was analyzed in the GSE20685 database. The result of the Kaplan–Meier analysis showed that BC patients in the pyroptosis-upregulated group had longer OS, DSS, and DFS (Figures 4E–G).

### Independent Prognostic Value of the Pyroptosis-Associated lncRNA Signature

**FIGURE 5:**
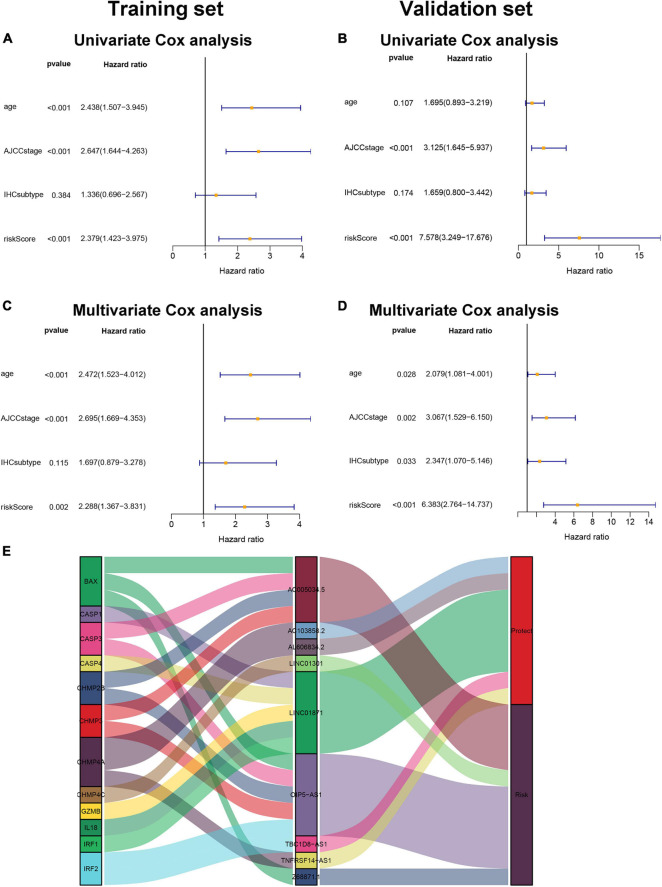
Pyroptosis-associated lncRNA signature is an independent prognostic factor. Univariate Cox regression analysis and multivariate Cox regression analysis of OS are performed in the training set **(A,C)** and the validation set **(B,D)**. **(E)**. LncRNA–mRNA coexpression relationship between the pyroptosis-associated lncRNAs and corresponding genes shown by Sankey diagram.

Univariate Cox regression analysis and multivariate Cox regression analysis were conducted to investigate the independent prognostic value of the pyroptosis-associated lncRNA signature for BC patients. As shown in Figures 5A,B, the risk score was a prognostic indicator for OS in both the training set and validation set (training set: hazard ratio (HR) = 2.379, 95% confidence interval (CI) = 1.423–3.975, *p* < 0.001; validation set: HR = 7.578, 95% CI = 3.249–17.676, *p* < 0.001). After including other confounders in the multivariate Cox regression analysis, the risk score was still an independent prognostic indicator for OS (training set: HR = 2.288, 95% CI = 1.367–3.831, *p* = 0.002; validation set: HR = 6.383, 95% CI = 2.764–14.737, *p* < 0.001; Figures 5C,D).

### The Relationship of lncRNA–mRNA Coexpression

The pyroptosis-associated lncRNA signature contained nine lncRNAs. In order to clearly demonstrate the prognostic value of these lncRNAs and their relationship with pyroptosis-associated genes, a Sankey diagram was constructed. As shown in Figure 5E, lncRNA AC005034.5 was coexpressed with four pyroptosis-related genes (BAX, CASP3, CHMP2B, and CHMP3), lncRNA LINC01871 had coexpressive relationship with five pyroptosis-related genes (CASP1, CASP4, GZMB, IL18, and IRF1), and lncRNA OIP5-AS1 had coexpressive relationship with five pyroptosis-related genes (BAX, CASP3, CHMP2B, CHMP3, and IRF2). Among the nine pyroptosis-associated lncRNAs, five lncRNAs were protective factors (AC103858.2, AL606834.2, LINC01871, TBC1D8-AS1, and TNFRSF14-AS1), and four lncRNAs were risk factors (AC005034.5, LINC01301, OIP5-AS1, and Z68871.1).

### Gene Set and Function Enrichment Analysis

**FIGURE 6:**
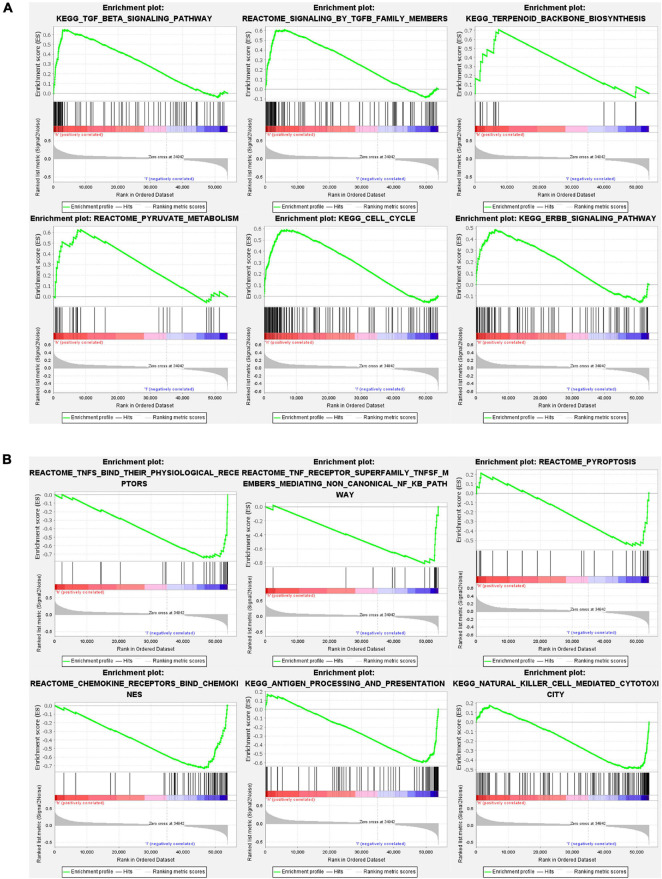
Gene set and function enrichment analysis of differentially expressed genes between the high-risk group and low-risk group. **(A)** TGF-β signaling pathway, terpenoid backbone biosynthesis, pyruvate metabolism, cell cycle, and ERBB signaling pathway are enriched in the high-risk group. **(B)** TNFs bind their physiological receptors, TNF receptor superfamily TNFSF members mediating non-canonical NF-kB pathway, pyroptosis pathway, chemokine receptors bind chemokines pathway, antigen processing and presentation pathway, and NK cell-mediated cytotoxicity pathway are enriched in the low-risk group.

**FIGURE 7:**
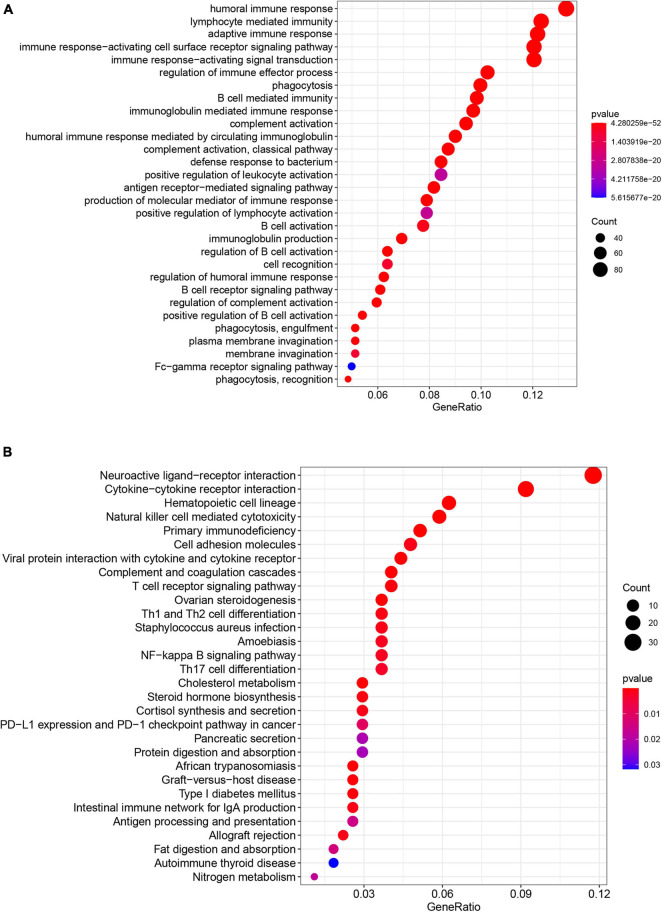
Results of GO enrichment analysis and KEGG pathway analyses. **(A)** GO enrichment analysis suggests that the differentially expressed genes between the low-risk and high-risk groups are mainly enriched in immune-associated biological processes. **(B)** KEGG pathway analysis suggests that the differentially expressed genes between the low-risk and high-risk groups were mainly enriched in immune-associated pathways.

Gene set and function enrichment analysis (GSEA) was conducted to investigate the signal pathways related to the pyroptosis-associated lncRNA signature. The results of the GSEA demonstrated that antipyroptosis pathways, antioxidant pathways, and cell growth pathways, such as transforming growth factor-β (TGF-β) signaling pathways, terpenoid backbone biosynthesis (González-Burgos and Gómez-Serranillos, 2012), pyruvate metabolism, cell cycle, and ERBB signaling pathway, were enriched in the high-risk group (Figure 6A). On the other hand, the pathways promoting pyroptosis were downregulated in the high-risk group, such as tumor necrosis factors (TNFs) bind their physiological receptor, TNF receptor superfamily TNFSF members mediating non-canonical NF-kB pathway (Wang Y. et al., 2020), and pyroptosis pathway (Figure 6B). Interestingly, the antitumor immune signaling pathways were significantly enriched in the low-risk group, including NK cell-mediated cytotoxicity pathway, chemokine receptors bind chemokine pathway, and antigen processing and presentation pathway (Figure 6B). To further investigate the biological processes associated with the pyroptosis-associated lncRNAs, GO enrichment analysis and KEGG pathway analysis were performed. The result of the GO enrichment analysis suggested that the differentially expressed genes between the low-risk and high-risk groups were mainly enriched in immune-associated biological processes, such as humoral immune response, lymphocyte-mediated immunity, and adaptive immune response (Figure 7A). The result of the KEGG pathway analysis suggested that the differentially expressed genes between the low-risk and high-risk groups were mainly enriched in immune-associated pathways, such as NK cell-mediated cytotoxicity pathway, cytokine–cytokine receptor interaction pathway, and primary immunodeficiency pathway (Figure 7B).

### Tumor Immune Microenvironment of BC

**FIGURE 8:**
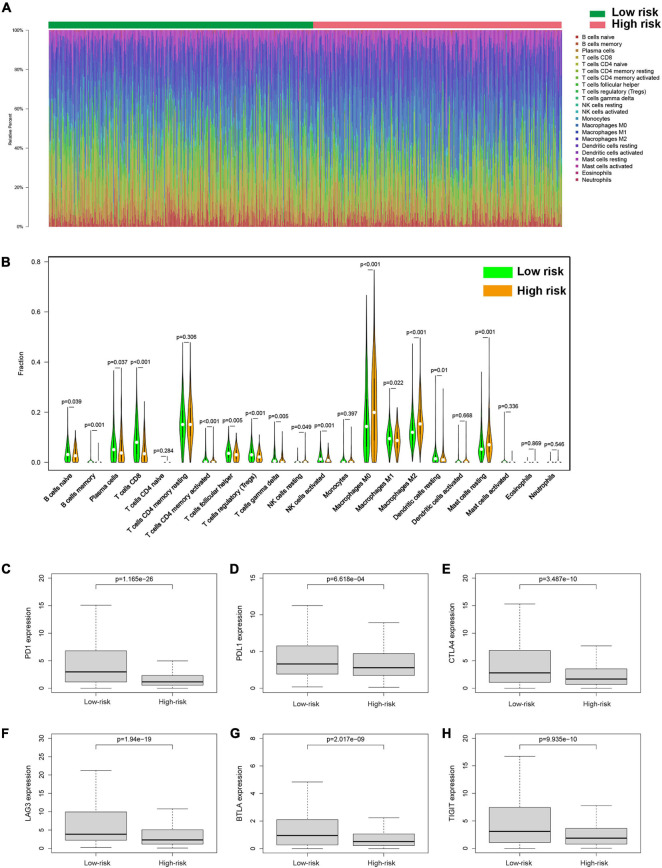
Tumor-infiltrating immune cells of BC patients. **(A)** The proportions of different tumor-infiltrating immune cells in the low-risk and high-risk groups. **(B)** Violin plot showed the different proportions of tumor-infiltrating cells between the high-risk group and low-risk group. The expression levels of PD1 **(C)**, PDL1 **(D)**, CTLA4 **(E)**, LAG3 **(F)**, BTLA **(G)**, and TIGIT **(H)** in the high-risk group and low-risk group.

**FIGURE 9:**
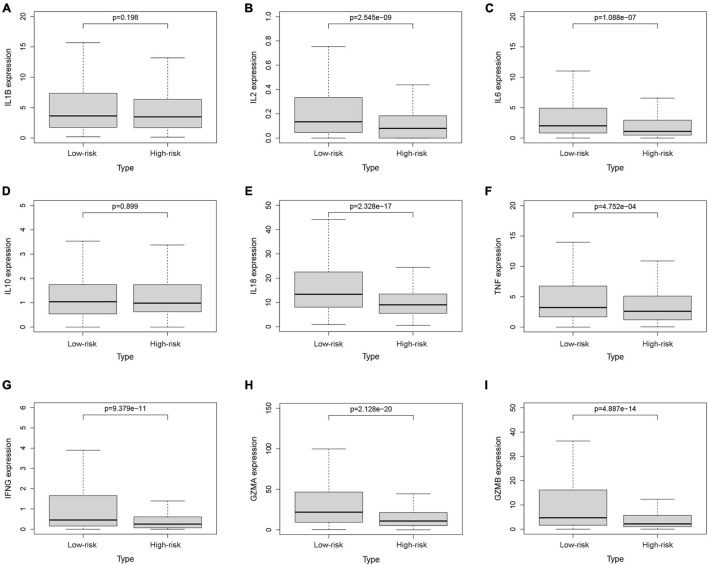
The expression levels of cytokines. The expression levels of IL-1β **(A)**, IL-2 **(B)**, IL-6 **(C)**, IL-10 **(D)**, IL-18 **(E)**, TNF **(F)**, IFN-γ **(G)**, GZMA **(H)**, and GZMB **(I)** between the low-risk and high-risk groups.

To investigate the relationship between pyroptosis-associated lncRNAs and tumor immune microenvironment (TME), the CIBERSORT algorithm was used to calculate the proportion of each kind of tumor-infiltrating immune cells in BC patients. The result showed that the proportions of different tumor-infiltrating immune cells between the low-risk and high-risk groups had a significant difference (Figure 8A). As shown in Figure 8B, the proportions of tumor-infiltrating B cell, CD8 + T cell, plasma cell, and activated NK cell were significantly lower in high-risk patients. However, the proportion of tumor-infiltrating M2 macrophages and mast cells was significantly higher in high-risk patients. Then, the difference in immune checkpoint molecules between the two groups was compared. As shown in Figures 8C–H, the expression levels of PD1, PDL1, CTLA4, LAG3, BTLA, and TIGIT were much higher in the low-risk group. The difference in expression level of cytokines between the high-risk and low-risk groups was also compared. The results showed that the expression levels of interleukin-2 (IL-2), IL-6, IL-18, TNF, interferon-γ (IFN-γ), GZMA, and GZMB were significantly higher in the low-risk group (Figures 9B,C,E–I), while there was no significant difference in the expression level of IL-1β and IL-10 (Figures 9A,D).

### The Pyroptosis-Associated lncRNA Signature in BC Patients Receiving Different Treatments

**FIGURE 10:**
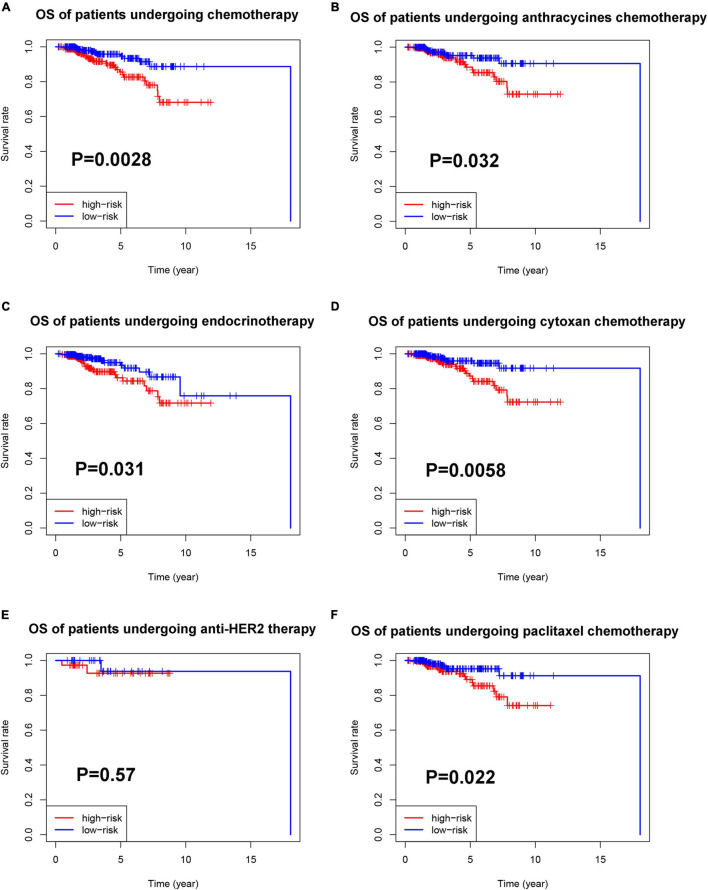
Prognostic value of the pyroptosis-associated lncRNA signature in patients receiving different treatments. **(A)** Kaplan–Meier curves for the OS of patients receiving chemotherapy between the high-risk and low-risk groups. Kaplan–Meier curves for the OS of patients receiving anthracycline **(B)**, cyclophosphamide **(D)**, and paclitaxel **(F)** between the high-risk and low-risk groups. **(C)** Kaplan–Meier curves for the OS of patients receiving endocrinotherapy between the high-risk and low-risk groups. **(E)** Kaplan–Meier curves for the OS of patients receiving anti-HER2 therapy between the high-risk and low-risk groups.

Then, we explored the prognostic value of the pyroptosis-associated lncRNA signature in BC patients receiving different treatment regimens. Among BC patients receiving chemotherapy, the patients in high-risk group based on pyroptosis-associated lncRNA signature had significantly poorer prognosis (Figure 10A). Among BC patients receiving endocrinotherapy, the patients in the high-risk group had poorer prognosis too (Figure 10C). The prognostic value of pyroptosis-associated lncRNA signature in patients undergoing anti-HER2 therapy was also analyzed, but there was no significant difference in prognosis of low-risk and high-risk patients (Figure 10E). To further investigate the value of pyroptosis-associated lncRNA signature in patients undergoing different chemotherapy regimens, the prognosis of patients treated with anthracycline, cyclophosphamide, or paclitaxel was analyzed, respectively. The results showed that patients in the high-risk group had poorer outcomes among patients treated with anthracycline, cyclophosphamide, or paclitaxel (Figures 10B,D,F).

### Construction and Validation of a Nomogram Based on the Pyroptosis-Associated lncRNA Signature

**FIGURE 11:**
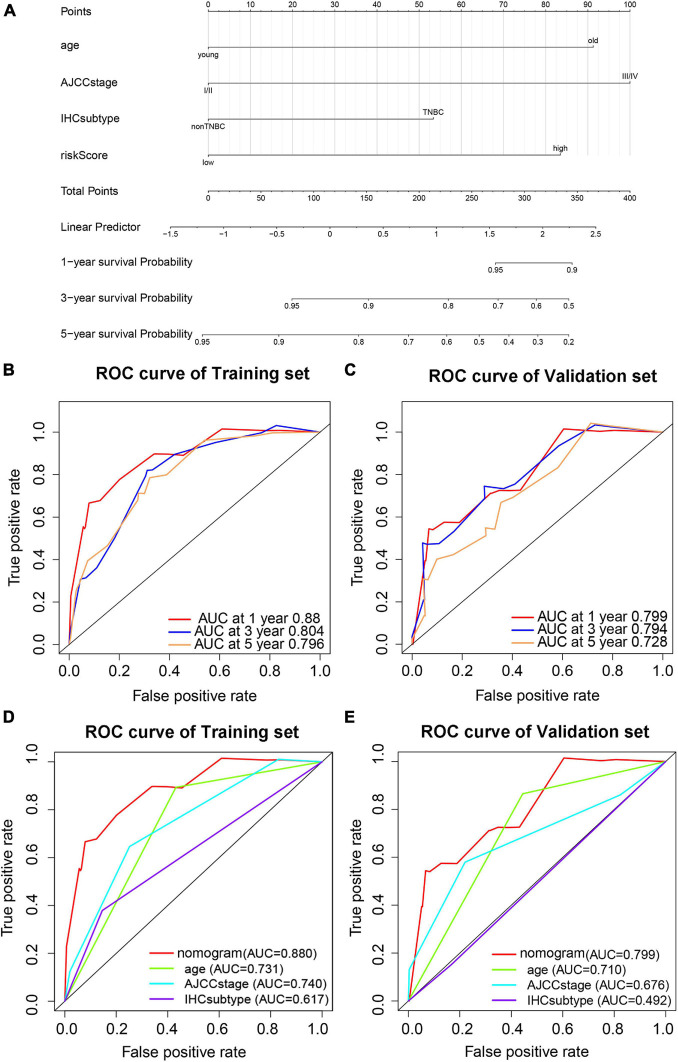
Prediction model based on pyroptosis-associated lncRNA signature for OS of BC patients. **(A)** Nomogram prediction model for OS of BC patients. Time-dependent ROC curves predict 1-year, 3-year, and 5-year OS in the training set **(B)** and validation set **(C)**. Predictive value of the nomogram and traditional prognostic indicators are compared by ROC curve analysis in the training set **(D)** and validation set **(E)**.

To provide a stable and accurate prediction model for BC patients, clinical features and risk score were included to construct a nomogram (Figure 11A). The predictive accuracy of the nomogram was evaluated by time-dependent ROC curve analysis; the result showed that area under the curve (AUC) of the nomogram in the training set was 0.880, 0.804, and 0.796 to predict 1-, 3-, and 5-year survival rates, respectively. In the validation set, it was 0.799, 0.794, and 0.728, respectively (Figures 11B,C). Then, the predictive values of the nomogram and traditional prognostic indicators, such as age, AJCC stage, and IHC subtype, were compared by ROC curve analysis. As shown in Figures 11D,E, the AUC of the nomogram was significantly higher than that of traditional prognostic indicators both in the training set and the validation set (Table 3).

**TABLE 3:** The AUC of nomogram, age, AJCC stage, and IHC subtype for prediction of OS in the training set and validation set.

	Training set		Validation set	
Factors	AUC (95% CI)	*p*-Value	AUC (95% CI)	*p-*Value
Nomogram	0.880 (0.850–0.907)		0.799(0.751–0.842)	
Age	0.731 (0.694–0.766)		0.710(0.656–0.759)	
AJCC stage	0.740 (0.703–0.774)		0.676(0.622–0.727)	
IHC subtype	0.617 (0.577–0.655)		0.492(0.472–0.536)	
Nomogram vs. age		0.019		0.4
Nomogram vs. AJCC stage		0.022		0.034
Nomogram vs. IHC subtype		0.002		0.02

## Discussion

Pyroptosis is a new type of programmed cell death characterized by the rupture of cell membranes and the release of inflammatory substances (Fang et al., 2020). In the development of cancer, the expression level of GSDMs in cancer cells is usually lower than that in normal tissues due to the high methylation of the promoters of pyroptosis-related genes, resulting in the growth and metastasis of tumors (Kim et al., 2008; Croes et al., 2017). Chemotherapy drugs and tamoxifen can inhibit the progression of tumors by inducing pyroptosis of tumor cells (Hu et al., 2020; Hwang and Chung, 2020). It has been proven that the expression level of GSDMs was closely related to the prognosis of BC (de Beeck et al., 2012). However, only a few lncRNAs related to pyroptosis have been reported at present, and a comprehensive analysis is needed.

In this study, nine optimal lncRNAs were identified for the pyroptosis-associated lncRNA signature. The genes coexpressed with these nine pyroptosis-associated lncRNAs were BAX, CASP1/3/4, CHMP2B/3, IRF1/2, GZMB, and IL-18. Among them, BAX is an important promoter of apoptosis and can also participate in pyroptosis. For example, navitoclax (a Bcl-2 inhibitor) can induce pyroptosis through the BAK/Bax-caspase3-GSDME signaling pathway (Hu et al., 2020). IRF2 is a transcription factor that directly regulates the expression levels of caspase-1 and caspase-4. When IRF2 is deficient, IRF1 can maintain the stable expression of caspase-4 with the presence of IFN-γ (Benaoudia et al., 2019). The nine-pyroptosis-associated lncRNA signature was proven to be an independent prognostic factor of BC patients. Then, a prediction model based on the pyroptosis-associated lncRNA signature was established. The AUC of the model for predicting OS could reach 0.880 in the training set and 0.799 in the validation set.

In order to further explore the relationship between pyroptosis and BC, functional enrichment analysis showed antipyroptosis, tumor metabolism, and cell cycle-related signaling pathways were enriched in the high-risk group, while the pyroptosis, antigen presentation, and NK cell-mediated cytotoxicity-related pathways were significantly activated in the low-risk group. TGF-β signaling is an important pathway in cancers and has both tumor-promoting and tumor inhibiting functions. It has been reported that TGF-β can suppress pyroptosis (Tamura et al., 2021). In this study, enrichment of TGF-β signaling pathway and TGF-β family members were observed in the high-risk group.

Moreover, more CD8 + T cells, activated NK cells, and activated CD4 + T memory cells were infiltrated in the TME of the low-risk group, suggesting a correlation between pyroptosis and TILs. In addition, patients in the low-risk group had a higher expression level of cytokines in the tumor tissues, such as IL-2, IL-6, and IL-18, which was consistent with more infiltrating immune cells in the low-risk group. The expression of TNF, IFN-γ, GZMA, and GZMB was higher in the low-risk group, suggesting a stronger cytotoxicity to tumor cells. At the same time, immunosuppressor molecules such as PD1, PDL1, CTLA4, LAG3, BTLA, and TIGIT in the low-risk group were significantly higher than those in the high-risk group. The increased infiltration of TILs and upregulated expression of immune checkpoint molecules suggested that patients in the low-risk group tended to be immunologically “hot” tumor, which was more likely to benefit from ICB therapy (Zhang and Chen, 2016).

Local inflammation caused by pyroptosis can lead to the formation of local immune escape (Kaplanov et al., 2019) and may be related to carcinogenesis (Wu et al., 2018). However, current studies have shown that the expression of GSDMs is positively correlated with the prognosis of cancer patients, and the efficacy of chemotherapy drugs and cytotoxic lymphocyte is partly dependent on pyroptosis (Wang Y. et al., 2017; Zhou et al., 2020). Pyroptosis is different from apoptosis. Theoretically, the former causes the death of cancer cells accompanied by the release of cytoplasmic contents, resulting in the exposure of TAAs, thus recruiting immune cells, such as NK cells, and CD8 + T lymphocytes to inhibit the growth of tumor. The conclusion drawn in this study is consistent with the theory and previous studies.

In this study, our pyroptosis-associated lncRNA signature could accurately predict the prognosis and tumor immune microenvironment of breast cancer patients. Low-risk patients not only had a better prognosis but also tended to be immunologically “hot” tumor, which was more likely to benefit from immune checkpoint inhibitors. Therefore, our signature could not only help clinicians accurately predict patients’ outcomes but also identify patients who were more suitable for immune checkpoint inhibitors. At the same time, we also found that high-risk patients had a poor prognosis and tended to be immunologically “cold” tumors, which was difficult to benefit from immune checkpoint inhibitors. How do we improve the prognosis of patients in the high-risk group? In mice models, bioorthogonal system to gasdermin (a technique that is able to control the release of active gasdermin in mice and selectively enter mouse tumor cells) could increase CD4+ and CD8+ cells in the TME of BC, while the percentage of CD4+ Foxp3+ regulatory T cells decreased, which showed a strong antitumor effect. At the same time, it could play a synergistic role with checkpoint blockade (Wang Q. et al., 2020), suggesting that pyroptosis could improve the effect of immunotherapy. Thus, we propose a combined regimen of immune checkpoint inhibitors and pyroptosis inducers for high-risk patients because pyroptosis inducers had the potential to recruit immune cells by inducing pyroptosis of cancer cells, and the immunologically “cold” tumor could turn into “hot” tumor, then the immune checkpoint inhibitor could be effective for high-risk patients.

However, our study had some limitations. This research data came from the TCGA public database, and basic experiments *in vivo* or *in vitro* will be conducted to confirm the efficacy of combined regimen of immune checkpoint inhibitors and pyroptosis inducers in the future. In addition, clinical trials are urgently needed to confirm whether inducing pyroptosis could improve the efficacy of immunotherapy in human BC patients.

In conclusion, we are the first to identify the pyroptosis-related lncRNAs associated with the prognosis of BC and establish a prognostic prediction model. At the same time, this study found that the pyroptosis risk score was related to TILs and the expression of immune checkpoint molecules. Thus, inducing pyroptosis may be a potential therapy to improve the efficacy of immunotherapy in BC.

## Data Availability Statement

Publicly available datasets were analyzed in this study. This data can be found here: The data of this study were downloaded from TCGA (https://portal.gdc.cancer.gov/repository).

## Author Contributions

XX, XQ, LP, and KZ participated in this research, including conception and design, drafting, and critical revision of the manuscript. KZ and XO acquired the data. LP and KZ analyzed the data and interpreted the data. LP and XO supported for material. XX and XQ supervised the study. All authors ensured and approved the final version.

## Conflict of Interest

The authors declare that the research was conducted in the absence of any commercial or financial relationships that could be construed as a potential conflict of interest.

## Publisher’s Note

All claims expressed in this article are solely those of the authors and do not necessarily represent those of their affiliated organizations, or those of the publisher, the editors and the reviewers. Any product that may be evaluated in this article, or claim that may be made by its manufacturer, is not guaranteed or endorsed by the publisher.

## Footnotes

1
https://portal.gdc.cancer.gov/repository

2
https://www.gsea-msigdb.org/gsea/msigdb/index.jsp

3
http://www.broadinstitute.org/gsea
